# *ELK4* transcription promotes *MSI2* ‐mediated progression of non‐small cell lung cancer through the *TGF‐β*/ *SMAD3* pathway

**DOI:** 10.1002/kjm2.12952

**Published:** 2025-02-19

**Authors:** Guo‐Cui Shi, Yu‐Qing Teng, Jin‐Song Zhu, Jia‐Wei Sun, Cui Liu, Yi‐Wei Zhang

**Affiliations:** ^1^ Department of Respiratory Medicine CANGZHOU People's Hospital Cangzhou Hebei China; ^2^ Outpatient Department, The Chinese People's Liberation Army Hebei Provincial Military Region Cangzhou Hebei China; ^3^ Graduate School Hebei Medical University Shijiazhuang Hebei China

**Keywords:** ELK4, MSI2, non‐small cell lung cancer, SMAD3, TGF‐*β*

## Abstract

Non‐small cell lung cancer (NSCLC) is a primary contributor to global cancer‐related mortality. Musashi‐2 (MSI2), an RNA‐binding protein (RBP), is upregulated in specific NSCLC tumor subgroups. The current investigation evaluated the role and underlying mechanism of MSI2 in NSCLC. The expression levels of ELK4, MSI2, SMAD3, p‐SMAD3 and TGF*β*R1 were assessed via RT–qPCR or Western blot. Chromatin immunoprecipitation (ChIP) and dual luciferase reporter assays were used to confirm the interaction between ELK4 and MSI2. The proliferation, migration and invasion of NSCLC cells were determined via MTT, colony formation, and transwell assays, respectively. A xenograft tumor model was established in BALB/c nude mice. Immunohistochemical (IHC) staining was used to test Ki67 expression. We found that MSI2 and ELK4 expression levels were increased in NSCLC tissues and cells. ELK4 depletion suppressed the proliferation, migration and invasion of NSCLC cells. ELK4 acts as a transcription factor and promotes the transcription of MSI2. MSI2 depletion repressed NSCLC cell proliferation, migration and invasion through the TGF‐*β*/SMAD3 pathway. Overexpression of ELK4 reversed the inhibitory effect of MSI2 repression on NSCLC progression. These results confirmed that ELK4 is a direct regulator of MSI2 expression and that MSI2 promotes NSCLC progression through TGF‐*β*/SMAD3 activation, suggesting the potential clinical value of inhibiting MSI2 in NSCLC.

**Abbreviations**
MTT: 3‐(4,5‐dimethylthiazol‐2‐yl)‐2,5‐diphenyltetrazolium bromide
CST: cell signaling technology
ChIP: chromatin immunoprecipitation
DAB: diaminobenzidine
DMSO: dimethyl sulfoxide
DMEM: Dulbecco's modified Eagle's medium
ELK4: ETS‐like transcription factor 4
FBS: fetal bovine serum
IHC: immunohistochemical staining
MSI2: Musashi‐2
NSCLC: non‐small cell lung cancer
RBP: RNA‐binding protein

## 1 INTRODUCTION

Lung cancer is a prevalent malignant tumor affecting the respiratory system and has become the leading cause of cancer‐related death in numerous countries.^1^ Non‐small cell lung cancer (NSCLC) is the most common type of lung cancer, accounting for 85% of all lung cancers.^2^ Despite the continuous improvement in medical standards, the diagnosis and treatment of NSCLC have not made significant progress in the past few decades. Most NSCLC patients are diagnosed at an advanced stage of the disease, which means that the tumor cells have metastasized or have the characteristics of infiltration and metastasis.^3^ The 5‐year survival rate of patients is less than 15%, and the prognosis is very poor.^4^ Therefore, an in‐depth understanding of the driving factors and their related mechanisms is crucial for improving the survival rate of NSCLC patients.

RNA‐binding proteins are important regulators involved in the regulation of RNA metabolism and gene expression.^5^ As an important molecule in hematopoietic cells, the RNA binding protein Musashi‐2 (MSI2) is a heterogeneous ribonucleoprotein that was first discovered in myeloid leukemia and is considered an important marker for maintaining the stemness of hematopoietic stem cells.^6^, ^7^ Studies have confirmed that MSI2 expression is increased in most solid tumors and is crucial for tumor proliferation.^8^, ^9^ High levels of MSI2 expression are associated with a poor prognosis and disease progression in NSCLC patients.^10^ Additionally, MSI2 promotes increased invasion of lung cancer cells by regulating the TGF*β*R1/SMAD3 signaling pathway, which can be used as an effective biomarker for the prognosis of NSCLC.^11^ However, the role and mechanism of MSI2 in NSCLC are relatively unknown.

We predicted that ELK4 is a transcription factor of MSI2 via JASPAR software and that there are binding sites in the promoter region of MSI2. The transcription factor ETS‐like transcription factor 4 (ELK4) belongs to the ternary complex factor subfamily of E‐26 domain transcription genes.^12^ In addition, ELK4 plays a key role in many cancers, and its overexpression is related to the malignant phenotype of colorectal cancer and gastric cancer.^13^, ^14^ ELK4 also participates in immune regulation by guiding the differentiation process of αβCD8^+^ T cells.^15^ However, the biological function and regulatory mechanism of ELK4 in NSCLC are still unclear.

Therefore, we speculate that ELK4 transcriptionally promotes MSI2 to regulate NSCLC progression through the TGF‐*β*/SMAD3 pathway, providing a potential novel target for NSCLC treatment.

## 2 MATERIALS AND METHODS

### 2.1 Clinical specimens

Ten pairs of lung tumor biopsy tissues and corresponding adjacent tumor biopsy tissues (5 cm from the tumor edge) were collected from NSCLC patients who underwent surgery in the hospital. The 10 tumor tissues used in our study were adenocarcinomas. The histopathological types were determined according to the WHO pathological staging criteria. All patients were confirmed via pathological examination. Written informed consent was obtained from all participating patients. None of the patients had undergone preoperative chemotherapy or radiotherapy. The tissue samples were frozen and stored at −80°C. The Ethics Committee of the hospital approved the experimental protocols for this study. The human ethical clearance number is AF/SC‐08/01.0.

### 2.2 Cell culture

Human normal lung epithelial cells (BEAS‐2B) and NSCLC cell lines (A549, HCC827, PC9, H1299, and H1975) were obtained from the Cell Bank of the Chinese Academy of Biological Sciences (Shanghai, China) and maintained in Dulbecco's modified Eagle's medium (DMEM)/F12 with 10% fetal bovine serum (FBS) (Gibco, Waltham, MA, USA), 100 U/mL penicillin G, and 100 mg/mL streptomycin at 37°C with 5% CO_2_.

### 2.3 Cell transfection

sh‐ELK4 and sh‐MSI2 (Gene‐Pharma, Shanghai, China) were transfected into A549 and H1975 cells with Lipofectamine 2000 (Invitrogen, Carlsbad, CA, USA) for 48 h to knock down gene expression. Sh‐NC was used as a control. The pcDNA3.1‐ELK4 (oe‐ELK4), pcDNA3.1‐MSI2 (oe‐MSI2) and pcDNA3.1 control vectors (oe‐NC) were also purchased from Gene‐Pharma (Shanghai, China) and transfected into A549 and H1975 cells with Lipofectamine 2000 (Invitrogen) based on the manufacturers' protocol for 48 h.

### 2.4 Quantitative real‐time PCR

Total RNA was extracted from NSCLC cells or clinical samples via TRIzol reagent (Invitrogen) and reverse transcribed via the PrimeScript RT Reagent Kit (Takara, Kyoto, Japan). RT–qPCR was performed via SYBR Premix ExTaq™ (Takara) on an ABI Prism 7900 sequence detection system (Applied Biosystems, CA, USA). The sequences of primers used were as follows: ELK4 F: 5′‐GAAGAAACTATCCAAGCTTTGGAGAC‐3′, R: 5′‐AGGGGCTCGGAGTCAGCAAGATG‐3′; MSI2 F: 5′‐ATCCCACTACGAAACGCTCC‐3′, R: 5′‐GGGGTCAATCGTCTTGGAATC‐3′. GAPDH was used as an endogenous control. The relative gene expression was calculated via the 2^−∆∆*Ct*^ method.

### 2.5 Western blot

Total protein was extracted from cells and tissues via RIPA lysis buffer (Beyotime, Shanghai, China), after which the protein concentration was detected via the BCA assay (Pierce, Rockford, IL, USA). Proteins were separated by SDS–PAGE. Following transfer to PVDF membranes (Millipore, Bedford, MA, USA), the proteins were blocked with 5% nonfat milk and incubated with the following antibodies overnight at 4°C: ELK4 (ab86002, 1:1000, Abcam, Cambridge, MA, USA), MSI2 (ab76148, 1:1000, Abcam), SMAD3 (#9513, Cell Signaling Technology, CST, Danvers, MA, USA), p‐SMAD3 (#9520, 1:1000, CST), and TGF*β*R1 (#3712, 1:1000, CST). After being rinsed with TBST, the membranes were incubated with an HRP‐conjugated secondary antibody (#7074, 1:1000, CST) for 1 h. The signal was detected with an enhanced chemiluminescence (ECL) detection kit (Beyotime) and quantified with ImageJ (National Institutes of Health, Bethesda, MD, USA). *β*‐actin was used as a loading control.

### 2.6 Dual‐luciferase reporter assay

We cloned the full‐length MSI2 promoter containing the mutant or wild‐type sequences into the pGLO4.10 vector (Promega, MWI, USA). These constructs were subsequently cotransfected with the ELK4 overexpression vector or a mock vector into NSCLC cells for 48 h via LipofectamineTM 2000 (Invitrogen, CA, USA). Luciferase activity was determined via the Dual‐Luciferase Reporter Assay System (Promega).

### 2.7 Chromatin immunoprecipitation (ChIP)

We utilized a Simple Chip Enzymatic Chromatin IP kit (CST) to examine the impact of ELK4 on MSI2 regulation. In brief, A549 and H1975 cells were treated with formaldehyde for 10 min to establish DNA–protein cross‐linking. Subsequently, sonication was performed on the cell lysates to generate chromatin fragments, which were then subjected to immunoprecipitation utilizing either an anti‐ELK4 antibody (ab86002, Abcam) or an anti‐IgG antibody (ab172730, Abcam) as a control. Protein A agarose beads (Millipore) were used to collect the complexes, which were subsequently washed to eliminate nonspecific binding. Finally, qPCR was used to detect the promoter sequences of the MSI2 gene.

### 2.8 Colony formation assay

Single‐cell suspensions were collected after trypsin digestion, and approximately 300 cells per well were seeded in six‐well plates (Corning, NY, USA). The plates were then incubated at 37°C until visible colonies formed. The cells in the plates were subsequently fixed with methanol and quantified via 0.5% crystal violet staining.

### 2.9 3‐(4,5‐dimethylthiazol‐2‐yl)‐2,5‐diphenyltetrazolium bromide (MTT) assay

Cellular proliferation was tested via the MTT assay. Cell seeding was performed on culture plates, followed by transfection with oligonucleotides or plasmids. After 12, 24, 48, 72, and 96 h of transfection, 20 μL of MTT solution (2.5 mg/mL, Promega) was added to each well. After 4 h of incubation at 37°C, 100 μL of dimethyl sulfoxide (DMSO) was added to each well. The absorbance was assessed at 590 nm via a microplate reader (BioTek, Winooski, VT, USA).

### 2.10 Transwell assay

Twenty‐four hours following transfection, 1 × 10^5^ NSCLC cells were seeded in the upper chambers containing 200 μL of serum‐free DMEM. The upper chambers consisted of polycarbonate membranes (8 μm pore size, Corning, Bedford, MA, USA), either uncoated or precoated with a Matrigel matrix, to simulate conditions for cell migration or invasion, respectively. In the lower chambers, 600 μL of complete DMEM (containing 10% FBS) was added, and the mixture was incubated for 24 h. Subsequently, the cells that migrated or invaded through the matrix membrane to the lower chambers were fixed and stained with 0.5% crystal violet. Finally, three random fields were counted via a light microscope (Olympus, Tokyo, Japan).

### 2.11 Nude mouse tumor formation experiment

BALB/C nude mice (4–5 weeks old, weighing 18–22 g) were obtained from the Shanghai Cancer Institute (Shanghai, China). The mice were maintained in a controlled environment with a 12‐h light/dark cycle and were provided with a standard rodent diet. The room temperature was maintained at a constant level. A549 or H1975 cells (1 × 10^6^ cells/mL) transfected with stably integrated sh‐NC + oe‐NC, sh‐ELK4, or sh‐ELK4 + oe‐MSI2 constructs were inoculated into the left axillary skin of nude mice (*n* = 4 mice/group). Tumor growth was monitored and recorded at weekly intervals, starting 1 week after inoculation. After 28 days, the mice were euthanized, and tissue samples were collected for further analysis. The animal study protocol was approved by the Animal Ethical and Welfare Committee of the hospital. The animal ethical clearance number is AF/SC‐08/01.0.

### 2.12 Immunohistochemical (IHC) staining

The tumor tissues were fixed, dehydrated via an ethanol gradient, embedded, and sectioned into 4 μm slices. The sections were subsequently incubated with primary antibodies against Ki‐67 (MA5‐14520, Invitrogen) overnight at 4°C. Next, the sections were incubated with secondary antibody (ab97080, Abcam) for 30 min at 37°C, after which diaminobenzidine (DAB) staining (R&D Systems, Minneapolis, MN, USA) was performed. Positive cells were photographed via a microscope (Olympus).

### 2.13 Statistical analysis

The data are expressed as the means ± SDs and were analyzed via GraphPad Prism 7 software (GraphPad, San Diego, CA, USA). Each experiment was repeated three times. Student's *t*‐test was used to analyze the differences between two groups. One‐way ANOVA was used to compare data among multiple groups. *p* <0.05 was used to determine statistical significance.

## 3 RESULTS

### 3.1 MSI2 and ELK4 expression was upregulated in NSCLC

**FIGURE 1:**
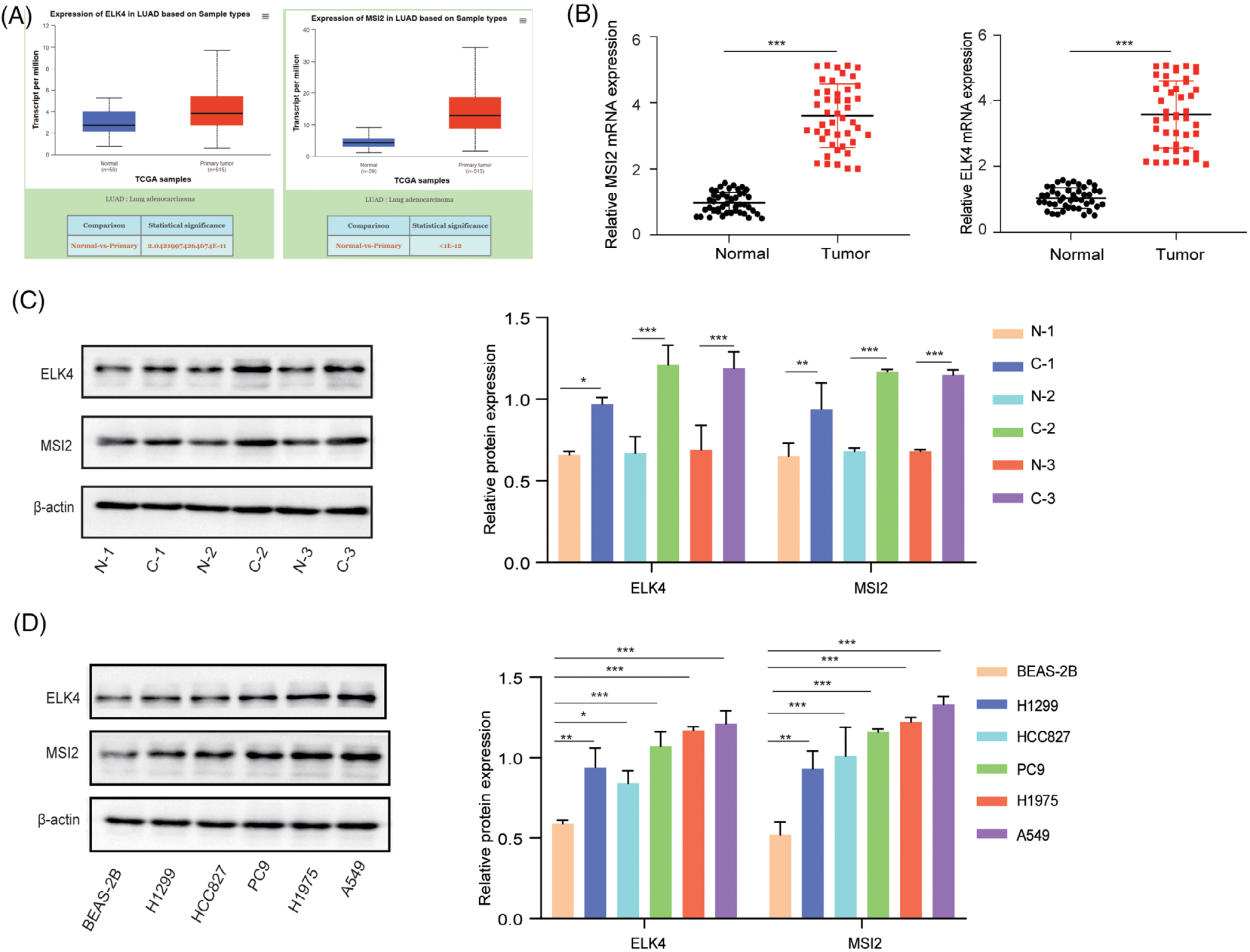
MSI2 and ELK4 expression was upregulated in NSCLC. (A) ELK4 and MSI2 expression in NSCLC tissues and adjacent normal tissues in the TCGA database. (B), (C) RT–qPCR and Western blot were used to analyze MSI2 and ELK4 levels in NSCLC tissues and normal paracancerous tissues. (D) MSI2 and ELK4 levels in BEAS‐2B cells and NSCLC cell lines (A549, HCC827, PC9, H1299, H1975) were determined via Western blot. The data are shown as the means ± SDs of three independent experiments. *p <0.05, **p <0.01, ***p <0.001.

First, the TGCA database results revealed that ELK4 and MSI2 levels were upregulated in NSCLC tissues compared with adjacent normal tissues (Figure 1A). Moreover, the mRNA and protein levels of MSI2 and ELK4 were significantly elevated in NSCLC tissues compared with normal paracancerous tissues (Figure 1B, C). Furthermore, compared with that in the human normal lung cell line BEAS‐2B, the expression of MSI2 and ELK4 was increased in NSCLC cell lines, especially in A549 and H1975 cells (Figure 1D). Overall, MSI2 and ELK4 levels are increased in NSCLC tissues and cells.

### 3.2 Downregulation of ELK4 repressed NSCLC cell proliferation, migration and invasion

**FIGURE 2:**
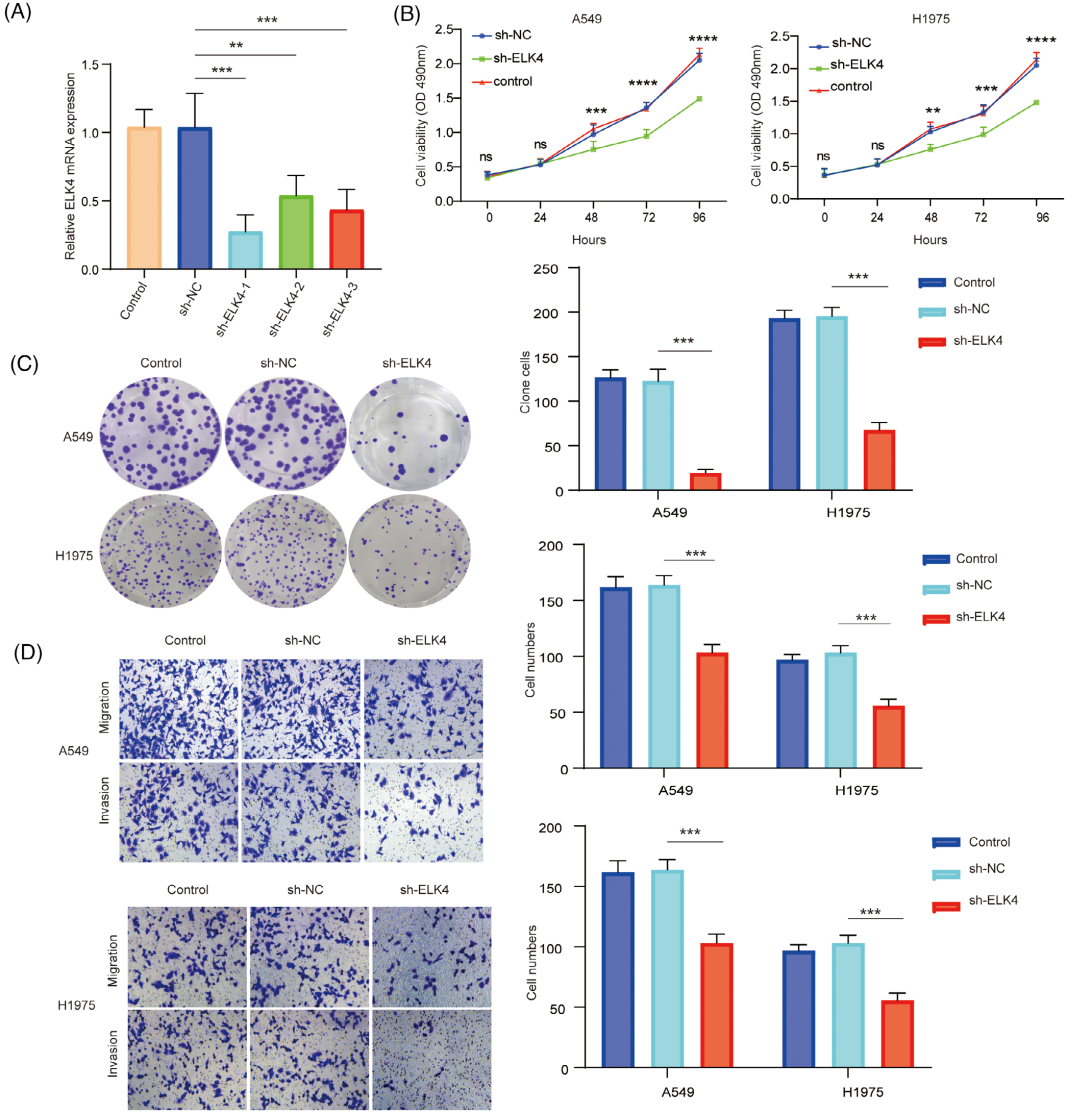
ELK4 downregulation repressed the proliferation, migration and invasion of NSCLC cells. A549 and H1975 cells were transfected with sh‐ELK4‐1, sh‐ELK4‐2, sh‐ELK4‐3 or sh‐NC. (A) The knockdown efficiency of sh‐ELK4 was determined via RT–qPCR. (B) Cell viability was tested via the MTT assay. (C) A colony formation assay was utilized to evaluate the effect of ELK4 depletion on cell proliferation. (D) The effects of ELK4 knockdown on migration and invasion were assessed via a transwell assay. All the data are presented as the means ± SDs of three independent experiments. *p <0.05, **p <0.01, ***p <0.001.

To evaluate the effect of ELK4 on the biological function of NSCLC cells, sh‐ELK4‐1, sh‐ELK4‐2, sh‐ELK4‐3 or sh‐NC was transfected into A549 and H1975 cells. ELK4 mRNA was successfully knocked down, and the knockdown efficiency of sh‐ELK4‐1 was the greatest (Figure 2A). MTT assays demonstrated that the knockdown of ELK4 inhibited the viability of NSCLC cells (Figure 2B). Moreover, ELK4 depletion suppressed the proliferation of NSCLC cells, as shown by colony formation assays (Figure 2C). In addition, transwell experiments revealed that the downregulation of ELK4 reduced the migration and invasion ability of NSCLC cells (Figure 2D). Therefore, these results demonstrate that ELK4 depletion may reduce NSCLC progression in vitro.

### 3.3 ELK4 acts as a transcription factor of MSI2 and promotes the transcription of MSI2

**FIGURE 3:**
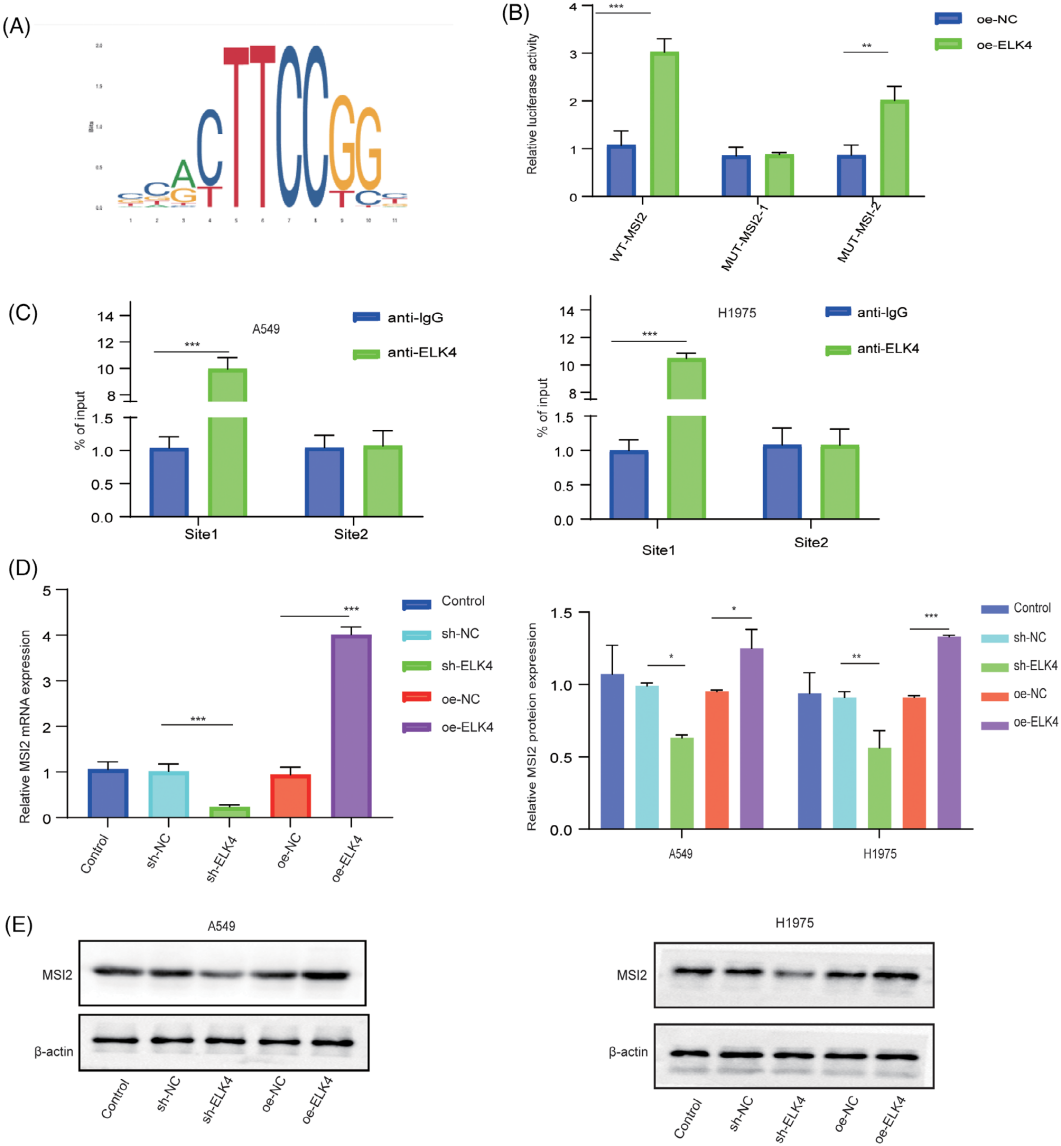
ELK4 acts as a transcription factor of MSI2 and promotes the transcription of MSI2. (A) The JASPAR database was used to predict the binding site of ELK4 to the promoter region of MSI2. (B) A dual‐luciferase assay was used to detect the binding between ELK4 and MSI2. (C) ChIP verified the binding of ELK4 to the MSI2 promoter region. (D), (E) A549 and H1975 cells were transfected with sh‐NC, sh‐ELK4, oe‐NC or oe‐ELK4. RT–qPCR and Western blot analysis of MSI2 levels. The error bars represent the means ± SDs of at least three independent experiments. *p <0.05, **p <0.01, ***p <0.001.

We further explored the intrinsic relationship between ELK4 and MSI2. The JASPAR database was used to predict the binding site of ELK4 to the promoter region of MSI2. A dual‐luciferase assay verified that MSI2 binds to ELK4 and that its ability to bind to site 1 (869–879) is the strongest (Figure 3A, B). Next, a ChIP assay revealed that ELK4 binds to site 1 of MSI2. (Figure 3C). Subsequently, sh‐NC, sh‐ELK4, oe‐NC or oe‐ELK4 was transfected into A549 and H1975 cells. ELK4 knockdown inhibited the mRNA transcription levels of MSI2, whereas ELK4 overexpression promoted the mRNA transcription levels of MSI2 (Figure 3D, E). Therefore, ELK4 transcriptionally induces MSI2 expression in NSCLC cells.

### 3.4 MSI2 knockdown inhibited the proliferation, migration and invasion of NSCLC cells through the TGF‐*β*/SMAD3 pathway

**FIGURE 4:**
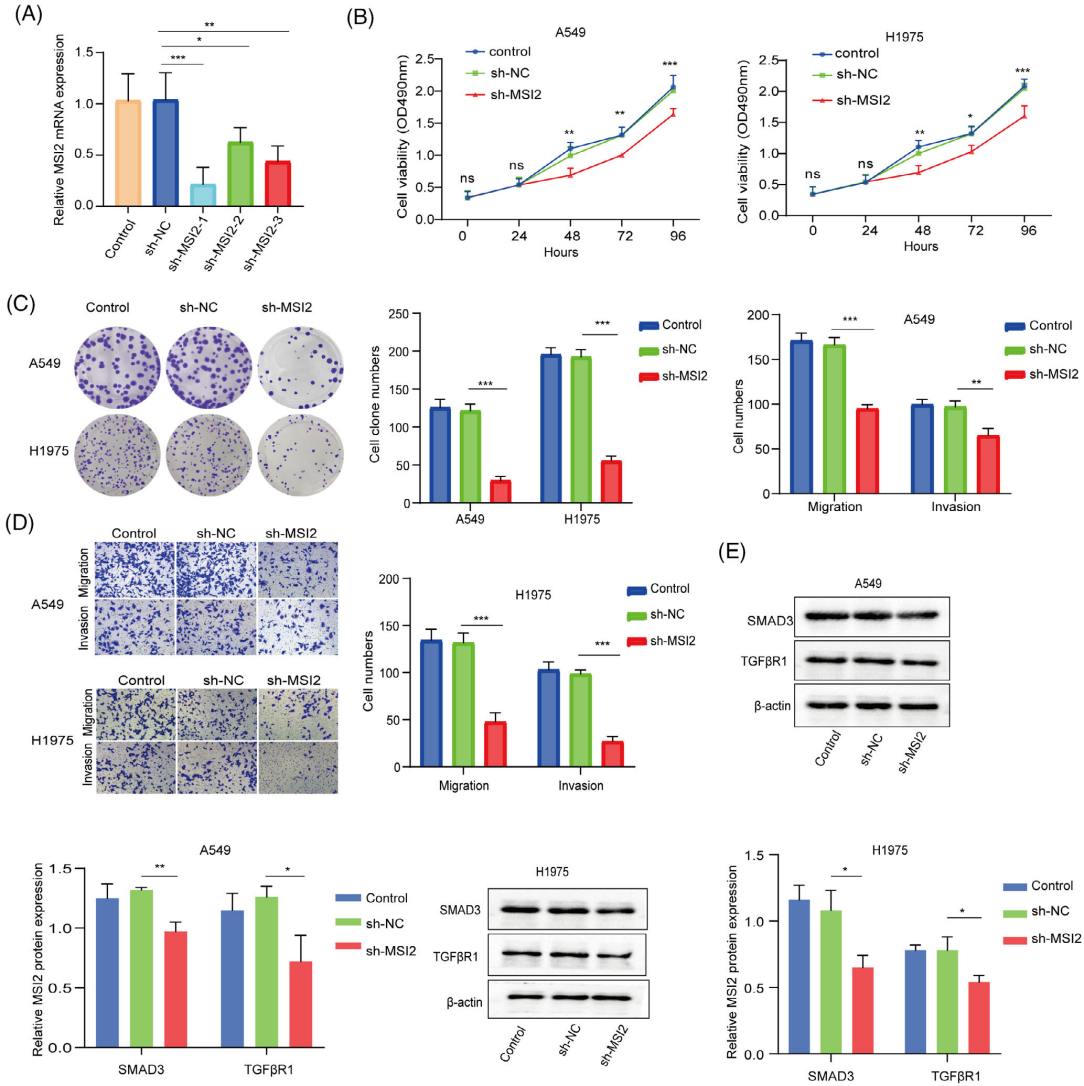
MSI2 knockdown inhibited the proliferation, migration and invasion of NSCLC cells through the TGF‐*β*/SMAD3 pathway. A549 and H1975 cells were transfected with sh‐MSI2 or sh‐NC. (A) The knockdown efficiency of sh‐MSI2 was determined via RT–qPCR. (B) Cell viability was tested via the MTT assay. (C) A colony formation assay was conducted to evaluate proliferation. (D) Cell migration and invasion were assessed via a transwell assay. (E) Western blotting was performed to detect SMAD3 and p‐SMAD3 protein levels. All the data are presented as the means ± SDs of three independent experiments. *p <0.05, **p <0.01, ***p <0.001.

Next, we investigated whether MSI2 regulates NSCLC progression in vitro. A549 and H1975 cells were transfected with sh‐MSI2 or sh‐NC. RT–qPCR analysis revealed that MSI2 was successfully knocked down, and sh‐MSI2‐1 had the highest knockdown efficiency for subsequent experiments (Figure 4A). Moreover, MSI2 deficiency reduced cell viability and proliferation (Figure 4B, C). Additionally, MSI2 repression inhibited the migration and invasion of NSCLC cells (Figure 4D). Notably, the Western blot results revealed that MSI2 downregulation reduced the protein expression of SMAD3 and TGF*β*R1 (Figure 4E). Thus, MSI2 silencing suppresses NSCLC progression in vitro through the TGF‐*β*/SMAD3 pathway.

### 3.5 Overexpression of MSI2 reversed the inhibitory effects of ELK4 knockdown on NSCLC progression

**FIGURE 5:**
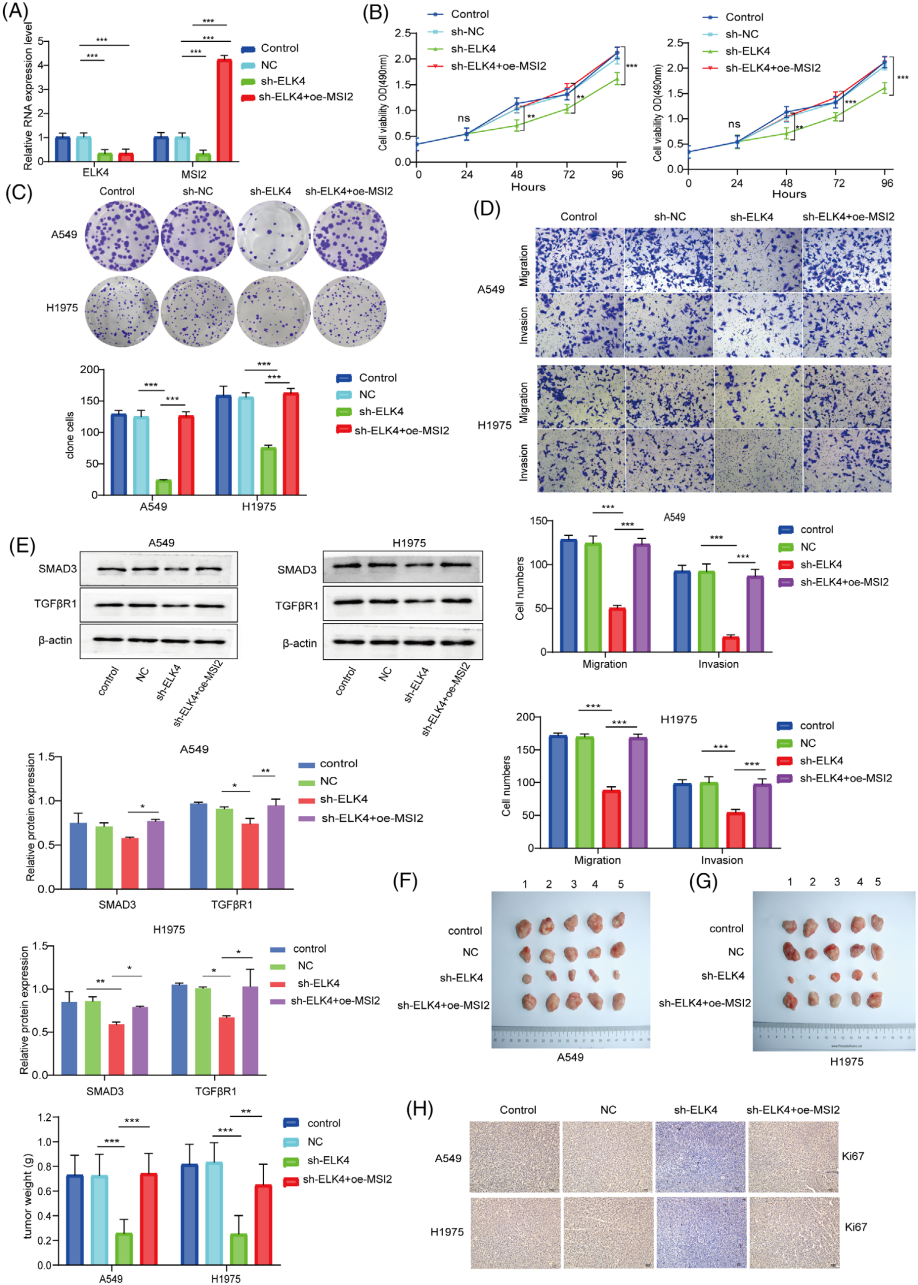
ELK4 overexpression reversed the inhibitory effects of MSI2 knockdown on NSCLC progression. sh‐ELK4 and oe‐MSI2 were transfected into A549 and H1975 cells. (A) The expression of ELK4 and oe‐MSI2 was determined by RT–qPCR. (B) Cell viability was tested via the MTT assay. (C) A colony formation assay was used to evaluate cell proliferation. (D) Cell migration and invasion were assessed via a transwell assay. (E) Western blot was used to detect SMAD3 and p‐SMAD3 protein levels. (F), (G) Images of xenograft tumors and analysis of tumor volume (*n* = 4 mice/group). (H) Ki67 levels in tumors were assessed via IHC. All the data are presented as the means ± SDs of three independent experiments. *p <0.05, **p <0.01, ***p <0.001.

We next explored whether ELK4 regulates NSCLC progression through MSI2. ELK4 silencing decreased the expression of ELK4 and MSI2, and the overexpression of MSI2 promoted MSI2 expression (Figure 5A). ELK4 knockdown repressed cell viability, proliferation, migration and invasion, whereas MSI2 upregulation reversed these effects (Figure 5B–D). Furthermore, the protein levels of SMAD and TGF*β*R1 in NSCLC cells were reduced by ELK4 knockdown, confirming that MSI2 relieved this inhibitory effect (Figure 5E). These results imply that the ELK4/MSI2 axis promotes NSCLC progression in vitro. Experiments in nude mice revealed that ELK4 silencing decreased tumor size, whereas MSI2 overexpression attenuated this inhibitory effect (Figure 5F, G). Moreover, the downregulation of ELK4 decreased Ki67 expression in tumor tissues, and the overexpression of MSI2 reversed this effect (Figure 5H). Therefore, these results demonstrate that the ELK4/MSI2 axis increases tumor formation in NSCLC in vivo.

## 4 DISCUSSION

Lung cancer remains the leading cause of cancer‐related death. Currently, lung cancer patients primarily receive combined therapies following surgery. As medical science and technology progress, molecular targeted therapy is increasingly emerging as the preferred option for cancer treatment, offering a new avenue for early intervention in NSCLC patients.^16^ Nonetheless, the 5‐year survival rate for most lung cancer patients is only 15%, with over 90% succumbing swiftly to lung cancer cell metastasis.^17^ Currently, an increasing number of researchers are focusing their efforts on the mechanisms and clinical treatment of this disease.^18^ Studies have reported a strong association between various carcinomas and RNA‐binding proteins.^19^ In this study, we investigated the potential role and mechanism of the RNA‐binding protein MSI2 in NSCLC progression and reported that ELK4 transcriptionally promoted MSI2 expression and regulated the progression of NSCLC through the TGF‐β/SMAD3 pathway. Hence, MSI2 plays a crucial role in the development of NSCLC through its oncogenic function.

ELK4 is a transcription factor belonging to the TCF subfamily of ETS domain transcription factors.^20^ ELK4, recognized as a proto‐oncogene, has been linked to the aggressive characteristics of glioblastoma, gastric cancer, and skin cutaneous melanoma.^21^, ^22^, ^23^ ELK4 plays a role in immune regulation by guiding differentiation processes in αβ CD8^+^ T cells.^15^ In NSCLC, ELK4 also serves as a crucial regulator. For example, AS‐tDR‐007333 accelerates NSCLC malignancy by stimulating ELK4 expression.^24^ In this study, we discovered that the ELK4 level was increased in NSCLC tissues and cells. In addition, ELK4 downregulation repressed the proliferation, migration and invasion of NSCLC cells. Notably, ELK4 could bind to the promoter region of MSI2 and positively regulate MSI2 expression. Therefore, these results demonstrate the significant contribution of ELK4 to NSCLC progression.

The MSI2 protein, which belongs to the Musashi protein family, serves as a crucial molecular marker of stem cells and early progenitor cells. Additionally, it is significantly associated with the prognosis and metastasis of diverse tumors.^25^, ^26^ In this study, we observed an increase in MSI2 levels in both NSCLC patients and cell lines. Furthermore, the downregulation of MSI2 significantly reduced the proliferation, migration, and invasion of NSCLC cells. MSI2 is an important regulatory factor in the development of NSCLC and may be closely related to the activation of TGF‐*β* signaling and the regulatory effect of MSI2 on oncogenes. For example, MSI2 plays a vital role in supporting TGF*β*R1/SMAD3 signaling, thereby contributing significantly to NSCLC progression.^11^ MSI2 depletion selectively impairs NSCLC cell proliferation by activating EGFR mutations.^27^ Additionally, cancer‐associated fibroblast‐derived MSI2 facilitates NSCLC metastasis by inducing epithelial–mesenchymal transition via paracrine IL‐6 signaling.^28^ Our results indicated that MSI2 downregulation reduced the protein levels of SMAD3 and TGF‐β. Additionally, the inhibitory effect of MSI2 knockdown on NSCLC progression in vitro and in vivo was reversed by ELK4 overexpression. Taken together, these results indicate that the ELK4/MSI2 axis promotes NSCLC progression by activating the TGF‐β/SMAD3 pathway.

In the present study, we detected increased expression of MSI2 and ELK4 in NSCLC tissues and cells. Clinical testing and in vitro experiments support the role of ELK4 in the transcriptional promotion of MSI2, thereby regulating NSCLC progression through the TGF‐β/SMAD3 pathway. Our findings suggest that both MSI2 and ELK4 possess oncogenic properties.^11^, ^24^ and exhibit promising potential as diagnostic and prognostic markers, as well as viable therapeutic targets, for NSCLC. Nevertheless, further studies are needed to validate this mechanism and explore its clinical application. Additionally, future studies are warranted to determine whether the ELK4/MSI2/TGF‐*β*/SMAD3 axis is involved in angiogenesis and epithelial mesenchymal transition in NSCLC.

## CONFLICT OF INTEREST STATEMENT

The authors declare that they have no conflicts of interest.

## Data Availability

All data generated or analysed during this study are included in this article.
